# Chronic and Acute Effects on Skin Temperature from a Sport Consisting of Repetitive Impacts from Hitting a Ball with the Hands

**DOI:** 10.3390/s22218572

**Published:** 2022-11-07

**Authors:** Jose Luis Sánchez-Jiménez, Robert Tejero-Pastor, María del Carmen Calzadillas-Valles, Irene Jimenez-Perez, Rosa Maria Cibrián Ortiz de Anda, Rosario Salvador-Palmer, Jose Ignacio Priego-Quesada

**Affiliations:** 1 Research Group in Sports Biomechanics (GIBD), Department of Physical Education and Sports, University of Valencia, 46010 Valencia, Spain; 2 Department of Physical Education and Sports, University of Valencia, 46010 Valencia, Spain; 3 Research Group in Medical Physics (GIFIME), Department of Physiology, University of Valencia, 46010 Valencia, Spain

**Keywords:** pelota valenciana, hand temperature, thermal image, sport, recovery pattern, cold stress test, thermoregulation

## Abstract

Valencian handball consists in hitting the ball with the hands and it may contribute to injury development on the hands. This study aimed to analyze skin temperature asymmetries and recovery after a cold stress test (CST) in professional players of Valencian handball before and after a competition. Thirteen professional athletes and a control group of ten physically active participants were measured. For both groups, infrared images were taken at the baseline condition; later they underwent a thermal stress test (pressing for 2 min with the palm of the hand on a metal plate) and then recovery images were taken. In athletes, the images were also taken after their competition. Athletes at baseline condition presented lower temperatures (*p* < 0.05) in the dominant hand compared with the non-dominant hand. There were asymmetries in all regions after their match (*p* < 0.05). After CST, a higher recovery rate was found after the game. The regions with the most significant differences in variation, asymmetries and recovery patterns were the index, middle and ring fingers, and the palm of the dominant hand. Taking into account that lower temperatures and the absence of temperature variation may be the consequence of a vascular adaptation, thermography could be used as a method to prevent injuries in athletes from Valencian handball.

## 1. Introduction

Valencian handball is a traditional sport of the Valencian Community (Spain) in which players must hit a ball of about 40–50 g with their hands [1]. To avoid injuries caused by repetitive hitting, the players protect their hands using special adhesive tape, metal plates and thimbles on almost all fingers except the thumbs [1,2]. However, Valencian handball athletes present a high injury incidence in the hands (approx. 65%) [2], with pain, callus, inflammation, bruising and Raynaud’s pathology being the most common [1,2].

Infrared thermography (IRT) is a non-invasive and non-harmful imaging technique that allows the assessment of skin temperature (Tsk) without contact [3,4]. This tool has been applicated in injury prevention in the sports field because Tsk asymmetries higher than 0.5 °C are associated with physiological dysfunction [5,6]. In this sense, processes such as inflammation or nerve dysfunction can affect Tsk [7,8] and therefore, it could be interesting to analyze Tsk asymmetries and Tsk recovery in the hands of Valencian handball players before and after playing a match. Moreover, to improve the diagnosis, dynamic thermography is applied for monitoring the Tsk recovery process after a thermal stress test (applying cold or heat) [9]. After a local cold exposure, the sympathetic response produces vasoconstriction and when the exposure ceases, during the rewarming, vasodilation occurs [10]. Therefore, alterations in vascular function could be assessed by using a cold stress test [11].

The hand Tsk of Valencian handball athletes has not been assessed previously, but other investigations with contact sports can be useful to understand the possible implications. In this sense, the ways that kickboxing and Muay Thai can alter Tsk asymmetries caused by repeated strikes were evaluated [12]. The results suggested that the side that is most exposed during fights, which usually receives the greater part of the strikes, presented higher Tsk (0.4C) in the shinbone and calf than the side less exposed [12]. However, no Tsk asymmetry was observed on the forearm in judo and jiu-jitsu athletes [12], which makes it difficult to hypothesize the chronic and acute effect of Valencian handball sport on Tsk asymmetries of the hands.

The aim of this study was to analyze Tsk asymmetries and recovery after a cold stress test in professional players of Valencian handball before and after a competition. We hypothesized that it is a sport that causes changes or physiological adaptations on the athletes’ hands that are perceived in the baseline Tsk or in the recovery of temperature after thermal stress, registered by IRT. Therefore, it was also hypothesized that there were greater asymmetries in Valencian handball players than in a physically active control population.

## 2. Materials and Methods

### 2.1. Participants

A sample size of 10 participants of each group (Valencian players and controls) was estimated using the G* Power 3.1 software (University of Düsseldorf, Düsseldorf, Germany) and considering a Student *T*-test, with 90% power, α value of 0.05 and an effect size of 1.4 for data on the baseline asymmetries of skin temperature, obtained by the analysis of the data of the first five participants of the study. Therefore, participants included a group of 13 professional male athletes of Valencian handball and a control group of 10 physically active men (Table 1). The participants of the control group performed sports that did not involve hitting with the hand, with the aim of discovering the chronic adaptations of the Valencian handball athletes. The professional athletes trained 6 ± 2 h per week, and they participated in two competitions each week during the season, achieving 3 ± 1 h of competition every week.

All the participants had the right hand as dominant, except for one from the athletes group. The main inclusion criteria for the athletes was that they played Valencian handball professionally within the Valencian Community. The inclusion criteria for the control group were that the men had an age range similar to the athletes and that they played a sport other than Valencian handball. The exclusion criteria of both groups were: that they had a diagnosis of hypertension and/or that they had suffered a hand injury in the last three months; and, in the case of the athletes, that the injury in their hands had deprived them from practicing Valencian handball. This research protocol was in accordance with the Declaration of Helsinki and was approved by the University of Valencia Ethics Committee (approval number H1550140204438).

### 2.2. Experimental Protocol

All members of the athletes group participated in a professional competition of Valencian handball sport and the control group did not play any match. The matches had a duration of 70 ± 11 min and they performed 9 ± 2 sets. The matches were recorded with a video camera for a posterior analysis of the number of hits that each player made with a dominant and non-dominant hand using the Dartfish EasyTag-Note application (version 2.0.11212.0, InMotion Technologies Ltd., Lausanne, Switzerland). The environmental conditions (recorded every 30 min) in the court were 26.9 ± 5.3 °C and 47 ± 12% relative humidity.

The matches were performed under the raspall modality (invasion type), which is characterized by the contact of the hands with the ground during the hitting due to the unlimited number of ball bounces on the ground.

All the measurements of athletes were conducted before and after the match. In addition, both before and after the match they performed the cold stress test (CST). The Control group was only assessed and performed the CST once, and therefore their data are only to compare the baseline condition. The information about the CST procedure is detailed in the Section 2.3.

Before and after the athletes’ competition their pain perception on the hands was evaluated using a visual analogue scale consisting of a 150 mm horizontal line [13]. A scale was made for each of the following four regions: right palm, right-hand fingers, left palm and left-hand fingers. The left end of the scale was labeled “no pain” and the right end “maximum pain imaginable”. 

### 2.3. Procedures

Tsk was assessed using an infrared thermal camera (FLIR E-60, Flir Systems Inc., Wilsonville, OR, USA) with a resolution of 320 × 240 pixels, a measurement uncertainty of ±2 °C or ±2% and a noise equivalent temperature difference <0.045 °C at 30 °C. The correct calibration of the camera was verified with a black body (BX-500 IR Infrared Calibrator, Shenzhen, China) before the experimental phase. All the measurements were conducted according to the latest methodology guidelines [14]. The camera was turned on 10 min before the first infrared image was taken and was placed at a distance of 1 m away from the participant, parallel to the corporal region of interest (hands) [14]. The reflected temperature was measured according to the standard method ISO 18434-1:2008 and introduced into the camera setup. All the images were indoor measurements without the influence of solar radiation and wind flow, as opposed to outdoor contexts with sun exposure [15]. Moreover, the measurements were conducted in a room under controlled temperature and relative humidity. The temperature and humidity in the room were measured with a digital thermohygrometer (TFA Dostmann, Wertheim-Reicholzheim, Germany) and imputed into the camera setup for every thermographic measurement. The environmental room conditions were 23.6 ± 3.6 °C and 53 ± 11% relative humidity. There were no infrared radiation source objects in the room [14]. The thermal stress indicator Wet-Bulb Globe Temperature was calculated resulting in 32.3 ± 1.7 °C [16].

To reduce the variability of Tsk, participants were instructed to avoid undergoing UV treatment, prolonged sunbathing, smoking, exhausting exercise, and consuming alcoholic, energy and/or caffeinated beverages in the 12 h before testing [14]. All the instructions were reiterated to the participants 24 h prior to the assessment and their accomplishments were verified on the day of the measurement.

**Figure 1:**
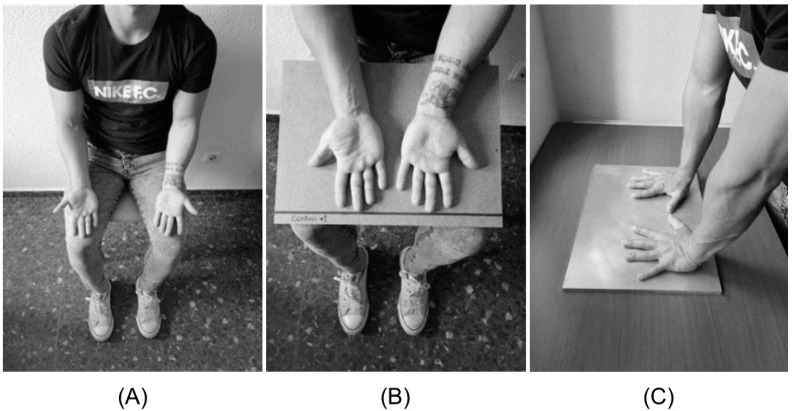
Different positions employed during the study. (**A**) Position to adapt the hands to environmental conditions. (**B**) Hand position during infrared imaging. (**C**) Cold stress test on a metal/aluminum plate.

Participants were in the measurement room for at least 10 min, sitting on a chair with their hands on the legs and palms up, to adapt to the environmental conditions (Figure 1A) [14]. To capture the images, the participants maintained this position but with a wooden board on their legs on which they placed their hands with the palms up, which was used as an indicator of the position in which the hands should be and to standardize the background (Figure 1B). The baseline image of the palms (pre-match measurement) was taken and then the CST was performed. 

The CST consisted of placing and pressuring the hands on an aluminum plate [17,18] for two minutes [19]. The images of the recovery of the palms were then taken every minute for 5 min after the CST ended [19] to monitor the hands’ rewarming pattern (Figure 1C). The plate was placed in the same measurement room for the correct stabilization of its temperature before the participants arrived. Up to this step, the same protocol was carried out for both groups. 

Then, the athletes played the match, protecting their hands as they did normally. At the end of the match, the protections of the hands and the residual gel were removed. Subsequently (approximately two minutes after the match), an image was made of the palms of the hands (post-match measurement), then the CST was conducted and images of recovery of the Tsk of the palms were taken every minute for 5 min. All images were taken by the same trained thermographic technician.

**Figure 2:**
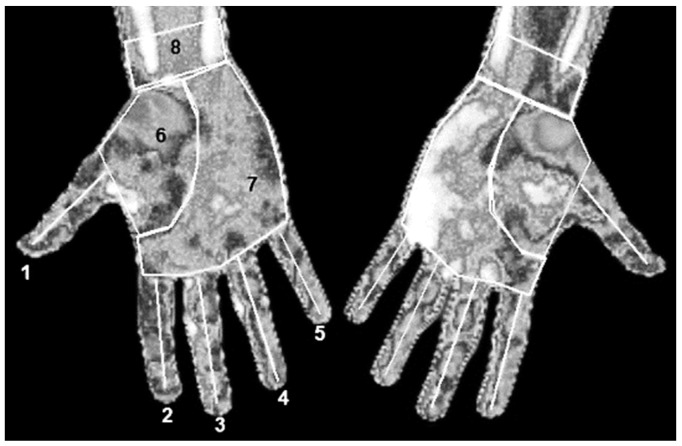
Regions of interest determined. (**1**) Thumb; (**2**) Index finger; (**3**) Middle finger; (**4**) Ring finger; (**5**) Little finger; (**6**) Thenar eminence; (**7**) Palm without thenar eminence; (**8**) Wrist.

The mean Tsk of 8 regions of interest (ROIs) of each hand (Figure 2) was obtained using thermography software (Thermacam Researcher Pro 2.10 software, FLIR, Wilsonville, OR, USA) and considering an emissivity of 0.98 [20]. Three variables were calculated: (1) Asymmetry: Tsk difference of each ROI between non-dominant and dominant hand, (2) Variation: Tsk difference of each ROI between the baseline and the post-match measurement moment of athletes, and (3) CST Variation: Tsk difference of each ROI between immediately after and each minute after CST.

### 2.4. Statistical Analysis

SPSS 21.0 (IBM Armonk, New York, NY, USA) was used for the statistical analysis. Data are reported as mean ± SD. The normality of the different variables was verified using the Shapiro-Wilk test (*p* > 0.05). To calculate differences between the control and the athlete group in the baseline condition for both hands, a Student *T*-test for related samples was applied. Repeated measures of ANOVA with Bonferroni post-hoc tests were applied in order to calculate the differences in asymmetry (dominant vs. non-dominant hand) in each of the three groups (control, athletes pre and athletes post). This same test was also used to calculate the differences in the variation in the athletes group (pre- vs. post-match) for each hand and between hands. To study the recovery pattern after the CST for each participant, ANOVAs with Bonferroni post-hoc tests were applied to assess the differences in the CST variation, i.e., the differences in each CST moment (immediately after vs. each minute until 5 min after CST) between the three groups (control vs. athletes pre vs. athletes post) for asymmetry, dominant hand and non-dominant hand. Pearson’s correlation coefficient analysis was used to assess the relationship between Tsk variation and other variables in athletes, such as the number of hits and hitting frequency, in the dominant hand. The significance level was set at *p* < 0.05. For significant pair differences, Hedge’s effect sizes (g) were computed and classified as small (g = 0.2–0.5), moderate (g = 0.5–0.8), or large (g > 0.8) [21]. 

## 3. Results

### 3.1. Thermal Asymmetry Analysis

**Figure 3:**
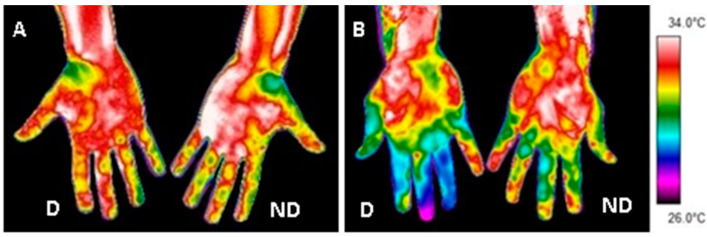
Example of the thermal images at baseline condition in a member of the control group (**A**) and a member of the athlete group (**B**). D = Dominant Hand; ND = Non-Dominant Hand.

Lower values of Tsk in the dominant hand at baseline condition were obtained in the athletes group than in the control group in the following ROIs: Index (*p* = 0.05; g = 0.6), Middle Finger (*p* = 0.01; g = 0.8), Ring Finger (*p* = 0.01; g = 0.8) and Little Finger (*p* = 0.03; g = 0.6) (Table 2) (Figure 3). However, no significant differences were obtained (*p* > 0.05) in Tsk in the non-dominant hand at baseline condition between groups. 

No significant differences (*p* > 0.05) were found between the Tsk of the dominant and non-dominant hands of the control group in any of the ROIs (Table 2). No significant differences were obtained (*p* > 0.05) in Tsk between the dominant and non-dominant hands in the Index, Little Finger and Wrist regions in the pre-match measurement in the athletes group. However, lower values of Tsk in the dominant hand were obtained in the remaining ROIs: Thumb (*p* = 0.005; g = 0.2), Middle Finger (*p* = 0.013; g = 0.4), Ring Finger (*p* = 0.009; g = 0.4), Thenar Eminence (*p* = 0.040; g = 0.2) and Palm (*p* = 0.039; g = 0.2). In the same line, after the match, the athletes group presented higher values of Tsk in the non-dominant hand in comparison with the dominant hand in all the regions: Thumb (*p* < 0.001; g = 0.5), Index (*p* = 0.004; g = 1.0), Middle Finger (*p* = 0.005; g = 0.9), Ring Finger (*p* < 0.001; g = 0.8), Little Finger (*p* = 0.030; g = 0.3), Thenar Eminence (*p* = 0.001; g = 0.7), Palm (*p* = 0.001; g = 0.6) and Wrist (*p* = 0.007; g = 0.4).

The athletes group reported higher Tsk in all the ROIs of the dominant and non-dominant hands after the match than before it (*p* < 0.05; g = 0.5–1.0), except Index and Middle Fingers of the dominant hand, which did not show differences in Tsk between these measurements (*p* > 0.05; g = 0.2–0.4).

**Figure 4:**
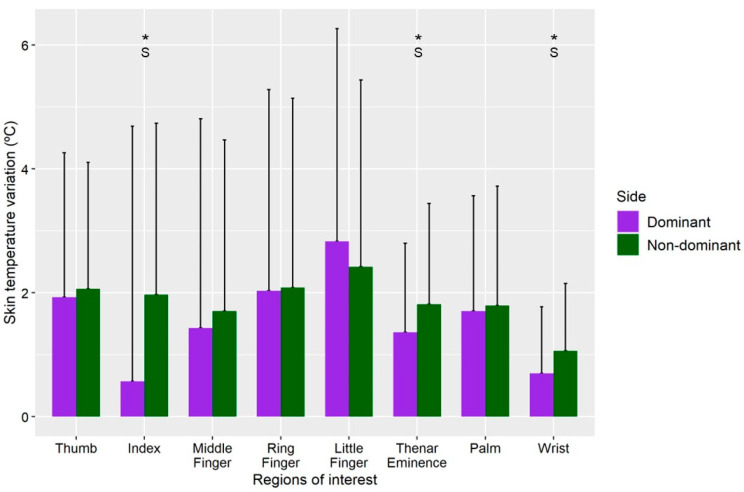
Mean and standard deviation of skin temperature variation in the dominant and non-dominant hand by ROIs in the athletes group. Differences were analyzed between the dominant and non-dominant hand (* *p* < 0.05). S = Small Effect Size.

Lower Tsk variations were obtained in all the ROIs in the dominant hand in comparison with the non-dominant hand, except the Little Finger which reported a higher Tsk variation in the dominant hand than in the non-dominant hand (Figure 4). However, Index, Thenar Eminence and Wrist were the only ROIs which reported statistically significant reductions in the Tsk variation between hands (*p* < 0.05; g = 0.3).

### 3.2. Thermal Stress Protocol

**Figure 5:**
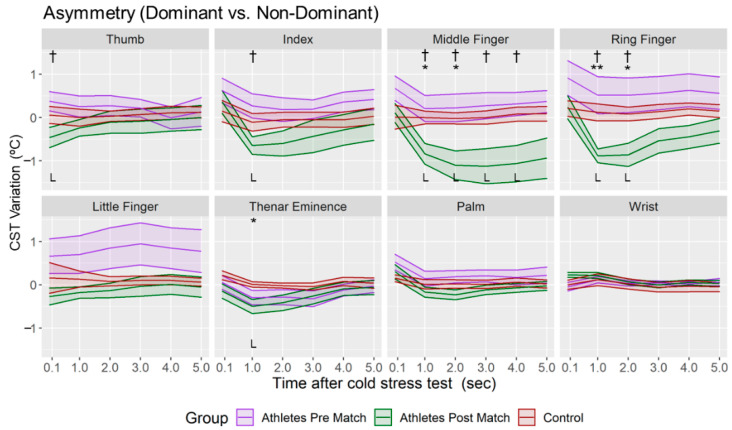
Mean and standard deviation of the CST variations of the asymmetries for each minute after CST in all the groups. L = Large Effect Size. Difference between Athletes Group—Post Match and Athletes Group—Pre Match († *p* < 0.05); Difference between Control Group and Athletes Group—Post Match (* *p* < 0.05; ** *p* < 0.01).

**Figure 6:**
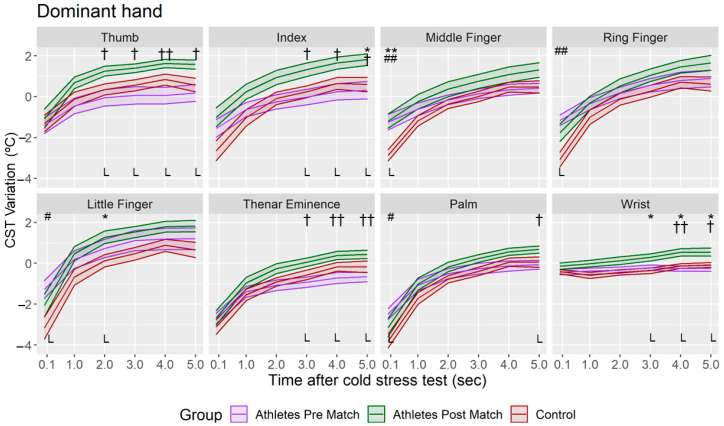
Mean and standard deviation of the CST variation in dominant hand for each minute after CST in all the groups. L = Large Effect Size. Difference between Athletes Group—Post Match and Athletes Group—Pre Match († *p* < 0.05; †† *p* < 0.01); Difference between Control Group and Athletes Group—Post Match (* *p* < 0.05; ** *p* < 0.01); Difference between Control Group and Athletes Group—Pre Match (# *p* < 0.05; ## *p* < 0.01).

**Figure 7:**
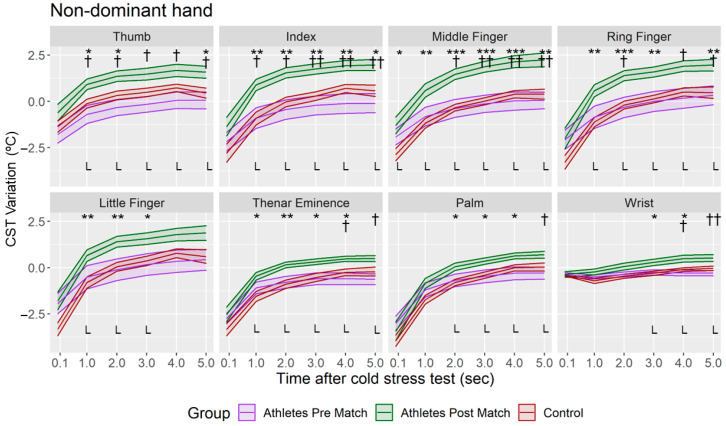
Mean and standard deviation of the CST variation in non-dominant hand for each minute after CST in all the groups. L = Large Effect Size. Difference between Athletes Group—Post Match and Athletes Group—Pre Match († *p* < 0.05; †† *p* < 0.01); Difference between Control Group and Athletes Group—Post Match (* *p* < 0.05; ** *p* < 0.01; *** *p* < 0.001).

The CST variation of the asymmetry was higher in the fingers than in Thenar, Palm and Wrist regions in all the groups (Figure 5). Negative and lower asymmetry variations (*p* < 0.05) were obtained when post-match measurements were compared with those of the pre-match group (in Thumb, Index, Middle and Ring fingers) and control group (in Middle and Ring fingers, and Thenar Eminence), especially in the first three minutes of the Tsk restoration. However, no differences were found between the pre-match group and control group (*p* > 0.05). According to the CST variation of the dominant and non-dominant hand (Figure 6 and Figure 7), it was higher in the post-match than control and pre-match groups in both hands. In addition, in only the dominant hand the CST variation was also higher in the pre-match group compared with the control group in the Middle, Ring and Little fingers and the Palm (*p* < 0.05) at the moment immediately after the CST.

### 3.3. Relationship between Tsk Variation and Hand Hitting Count

The mean and standard deviation of hand hitting performed by the athletes group in a match was 141 ± 53 (dominant hand, 124 ± 51 (87%); non-dominant hand, 17 ± 8 (13%)). Only one significative and inverse correlation (*p* = 0.004; r = −0.74) was found on Tsk variation in the Palm ROI with frequency of hand hitting (hitting × minute) (Table 3). However, no significant correlations (*p* > 0.05) were obtained in the remaining ROIs when these were correlated with frequency of hitting and percentage of dominant hand hitting. 

## 4. Discussion

The objective of this study was to analyze Tsk asymmetries and recovery after a cold stress test in professional players of Valencian handball before and after a competition. The main finding of this study is that the athletes group showed lower baseline Tsk than the control group in four of the ROIs assessed (Index, Middle Finger, Ring Finger and Little Finger). Moreover, playing a match of Valencian handball led to an acute increase in their Tsk asymmetries. Baseline Tsk restoration after the cold stress test did not differ between athletes and the control group, but this Tsk restoration increased after the match in the athletes group. Finally, an inverse correlation was observed between Tsk variation in Palm ROI and frequency of hand hitting during the match.

### 4.1. Thermal Asymmetry Analysis

Valencian handball is characterized by hitting a ball with the hands and receiving impacts. For this reason, the habitual practice of this activity may induce physiological adaptations in the hands. We obtained lower baseline Tsk in four ROIs of the dominant hand (Index, Middle Finger, Ring Finger and Little Finger) in the athletes group in comparison with the control group, supporting the hypothesis that Valencian handball produces changes or adaptations in athletes’ hands. These fingers are involved in specific Valencian handball skills of the modality assessed and the athletes employ protections in the fingers to avoid injury development [22]. These results suggest that the hands which receive impacts adapt to this situation, showing an effect on Tsk. Moreover, one of the most reported injuries in Valencian handball athletes is a painful callus produced by incorrect hitting or an overload of impacts in the same location [1] and this skin alteration may affect Tsk. However, the athletes included in the study had not suffered any injuries in the previous three months. In this sense, the possible explanation is the fact that in another sport called Basque pelota, which is similar to Valencian handball, these fingers are often affected by a white finger injury, in which the temperature restoration on the fingers is comparable with that in people affected by Raynaud syndrome [23]. 

Concerning Tsk asymmetries between the dominant and non-dominant hand, the lack of differences in baseline Tsk between the dominant and non-dominant hand in the control group supports the hypothesis that there is no presence of pathological conditions [24,25]. However, the athletes group presented lower Tsk in five of the ROIs (Thumb, Middle Finger, Ring Finger, Thenar Eminence and Palm) in the dominant hand in comparison with the non-dominant hand. In this sense, these results confirm the hypothesis that athletes have greater asymmetries than the control group. This asymmetric Tsk distribution between both hands may be produced by the chronic effect of previous injuries, because 76.5% of professional athletes have suffered an injury in the dominant hand [2]. In addition, this identification of asymmetries on Tsk in baseline conditions could be due to physiological adaptations in the dominant hands of the athletes. Tsk reduction may be due to the alteration of hand capillaries, which disturbs the blood flow on the hand produced by the repeated hitting, because chronic injuries have negative effects on microcirculation [26], similarly to repetitive hand exercise which results in a reduction in radial artery diameter at the wrist of the exercised hand [27]. In this sense, in pig slaughterhouse employees, lower Tsk was also observed in the hands most frequently used to manipulate the carcasses [28].

In our study, we observed higher Tsk in both hands after the match in the athletes group. In this sense, although this Tsk increase could be associated with inflammation due to the hits, the index and middle finger of the dominant hand did not present differences in Tsk between pre- and post-match measurements, despite the second and third metacarpal heads being the regions which receive the major impact pressures during hitting in Valencian handball [22]. To continuously subject these fingers to elevated pressures and contact with the ground during the hitting may be the reason for the development of an alteration in blood vessels. Moreover, with the aim to prevent injuries, Valencian handball athletes wear hand protection, and sport garments which cover the skin can affect Tsk due to the insulation of the garment and a reduced effectivity of sweat evaporation [29].

The Tsk variation supported the increment in Tsk after the match in all the ROIs. However, three ROIs (Index Finger, Thenar Eminence and Wrist) reported differences in Tsk variation between both hands, obtaining lower Tsk variation in the dominant hand in comparison with the non-dominant hand. These results suggest that the effect of playing a match mainly affects Tsk in the dominant hand and therefore it may contribute to the possible physiological adaptations observed in baseline values. However, the wrist presented an asymmetry in Tsk between both hands, despite this area not making contact with the ball and the floor. One possible explanation may be that a reduction in finger or palm blood flow, produced by a physiological adaptation, also affects wrist blood flow. Finally, concerning the effect of the Valencian handball match on Tsk asymmetries, all the ROIs assessed showed lower values of Tsk in the dominant hand than in the non-dominant hand. However, only five ROIs presented asymmetries before the match, suggesting that the match had an acute effect on Tsk. 

### 4.2. Thermal Stress Protocol

A cold stress test was performed to identify differences in Tsk restoration between Valencian handball athletes and the control group. Tsk restoration to baseline values was assessed during five minutes, but no differences were observed in hand skin temperature immediately after CST and during the first minute. These results may be produced by the effect of different intrinsic factors dependent on the individuals (circadian rhythms, metabolic rate or different skin blood flow) [30]. However, five of the ROIs of the dominant hand achieved the starting values in the third minute, especially in the athlete group, proving the hypothesis established previously, which was that playing Valencian handball produces adaptations in athletes’ hands and it affects Tsk restoration after the thermal stress test. In this sense, the results obtained were similar to those obtained by Binzoni et al. [31] who reported that three minutes of reperfusion is enough to return to baseline condition after a thermal stress test [31]. However, in our study, the wrist region did not show a high correlation in Tsk restoration, probably due to this region not being in contact with the metal plate during the cold stress test. All the restoration patterns of the hand obtained a high correlation (r^2^ > 0.85) in all the groups, and this similarity between subjects of each group suggests that changes in the restoration pattern of the Tsk may be an indicator for injury prevention. 

### 4.3. Relationship between Tsk Variation and Hand Hitting Count

During a Valencian handball match, the hands receive a number of impacts in different ways, and these impacts may be the cause of chronic adaptations in the hands. However, no significant correlations (*p* > 0.05) were obtained when the variation of Tsk was related to the percentage of hitting conducted with the dominant hand and the frequency of hitting of the dominant hand. Only the Palm ROI reported an inverse correlation (*p* < 0.01; r = −0.74) with the frequency of hitting. One possible explanation may be that receiving impacts with a high frequency on the hands could make blood flow restoration difficult after each impact and it may reduce the changes in Tsk. 

### 4.4. Limitations of the Study and future studies

One of the limitations of the study is the sample size, due to the low number of Valencian handball practitioners. Moreover, the lack of awareness of the pressure conducted against the plate during the CST made it difficult to know if the test was performed in the same way by all members of the athletes group. In relation to the ambient conditions, the fluctuation in the room temperature between participants may affect the hands’ skin temperature due to peripheral blood flow variation. It is recommended in future studies to conduct a time-span study to monitor the acute and chronic effects of sport activities and to include women due to the presence of thermoregulatory and anatomical differences between sexes [32]. 

## 5. Conclusions

The lower Tsk observed in the athletes group in baseline condition in comparison with the control group suggests the presence of adaptations in the hands of the athletes. Moreover, the match had an acute effect on the athletes’ hands, increasing Tsk and Tsk restoration after CST. In addition, the negative correlation between the frequency of hitting and Tsk changes supports the hypothesis that hitting affects the blood vessels of the hand. In this sense, infrared thermography seems to be an interesting tool for injury prevention in Valencian handball. 

## Figures and Tables

**Table 1:** Mean ± standard deviation of characteristics of participants.

	Athletes Group (n = 13)	Control Group (n = 10)	*p*-Value
**Age (years)**	24 ± 4	26 ± 3	0.27
**Height (m)**	1.80 ± 0.05	1.78 ± 0.04	0.15
**Body mass (kg)**	80.7 ± 6.9	75.2 ± 8.2	0.95
**BMI (kg/m^2^)**	24.91 ± 1.87	23.70 ± 2.40	0.25
**Blood pressure (mmHg)**	122/77 ± 9/7	120/75 ± 9/8	0.61/0.83

BMI = Body Mass Index.

**Table 2:** Absolute values of skin temperature in dominant and non-dominant hands of all the groups.

	ROI	Dominant Hand		Non-Dominant Hand	
		Mean (°C)	SD (°C)	Mean (°C)	SD (°C)
**Control Group**	Thumb	32.82	1.45	32.83	1.25
	Index	32.75 *	1.41	32.68	1.29
	Middle Finger	32.67 *	1.47	32.60	1.24
	Ring Finger	32.66 *	1.46	32.77	1.32
	Little Finger	32.75 *	1.42	32.73	1.40
	Thenar Eminence	32.51	1.40	32.37	1.35
	Palm	33.01	1.13	32.91	1.19
	Wrist	32.89	1.06	32.72	0.97
**Athletes Group** **Pre Match**	Thumb	30.76	3.43	31.58 ^††^	3.12
	Index	30.34	3.74	31.09	4.00
	Middle Finger	29.04	4.22	30.77 ^†^	3.99
	Ring Finger	28.92	4.20	30.83 ^††^	4.01
	Little Finger	29.81	4.27	30.78	4.14
	Thenar Eminence	32.11	1.97	32.47 ^†^	2.08
	Palm	31.68	2.45	32.26 ^†^	2.46
	Wrist	32.65	1.17	32.75	1.35
**Athletes Group** **Post Match**	Thumb	32.7 ^#^	1.8	33.6 ^## †††^	1.8
	Index	30.9	2.2	33.1 ^# ††^	1.8
	Middle Finger	30.5	2.4	32.5 ^# ††^	2.1
	Ring Finger	31.0 ^#^	2.5	32.9 ^# ††^	1.8
	Little Finger	32.6 ^#^	2.0	33.2 ^# †^	2.3
	Thenar Eminence	33.5 ^##^	1.3	34.3 ^# ††^	1.0
	Palm	33.4 ^##^	1.1	34.1 ^## ††^	1.1
	Wrist	33.4 ^#^	1.1	33.8 ^## ††^	1.3

Difference between Athletes Group—Pre Match and Control Group (* *p* < 0.05); difference between dominant and non-dominant hand (^†^ *p* < 0.05; ^††^ *p* < 0.01; ^†††^ *p* < 0.001); difference between Athletes Group—Post Match and Athletes Group Pre Match (^#^ *p* < 0.05; ^##^ *p* < 0.01).

**Table 3:** Correlation between variation of skin temperature of the ROIs and dominant hand ball hitting.

		Thumb	Index	Middle Finger	Ring Finger	Little Finger	Thenar Eminence	Palm	Wrist
**% Hitting**	**Pearson Correlation**	−0.285	−0.137	0.003	0.051	−0.069	−0.056	−0.330	−0.152
	**Bilateral Significance**	0.344	0.655	0.992	0.869	0.824	0.855	0.271	0.620
**F Hitting**	**Pearson Correlation**	−0.130	−0.140	0.108	−0.299	−0.328	−0.425	−0.740	−0.184
	**Bilateral Significance**	0.673	0.648	0.725	0.321	0.274	0.148	0.004 **	0.547

(** *p* < 0.01); % Hitting = Percentage of Dominant Hand Hitting; F Hitting = Frequency of Dominant Hand Hitting (hitting × minute). Significant values (*p* < 0.05) are highlighted in bold.

## Data Availability

The datasets generated and analyzed during the current study are available from the corresponding authors on reasonable request.
